# Fbxl17 is rearranged in breast cancer and loss of its activity leads to increased global *O*-GlcNAcylation

**DOI:** 10.1007/s00018-019-03306-y

**Published:** 2019-09-27

**Authors:** Bethany Mason, Susanne Flach, Felipe R. Teixeira, Raquel Manzano Garcia, Oscar M. Rueda, Jean E. Abraham, Carlos Caldas, Paul A. W. Edwards, Heike Laman

**Affiliations:** 1 Department of Pathology at Tennis Court Road, University of Cambridge, Cambridge, CB2 1QP UK; 2 Hutchison-MRC Research Centre, Addenbrooke’s Site, Hills Road, Cambridge, CB2 0XZ UK; 3 Department of Oncology, Cancer Research UK Cambridge Institute and Cancer Centre, Li Ka Shing Centre, University of Cambridge, Cambridge, CB2 0RE UK; 4 Cambridge Breast Unit, NIHR Cambridge Biomedical Research Centre and Cambridge Experimental Cancer Medicine Centre at Cambridge University Hospitals NHS Foundation Trust, Cambridge, CB2 2QQ UK; 5 Present Address: Department of Otolaryngology and Head & Neck Surgery, Hospital of the Ludwig-Maximilians-University, Munich, Germany; 6 Present Address: Department of Genetics and Evolution, Federal University of São Carlos, São Carlos, São Paulo Brazil

**Keywords:** FBXL17, Genome rearrangements, *O*-GlcNAcylation, *O*-GlcNAc, UAP1, Ubiquitin, Phosphorylation, Breast cancer

## Abstract

In cancer, many genes are mutated by genome rearrangement, but our understanding of the functional consequences of this remains rudimentary. Here we report the F-box protein encoded by *FBXL17* is disrupted in the region of the gene that encodes its substrate-binding leucine rich repeat (LRR) domain. Truncating Fbxl17 LRRs impaired its association with the other SCF holoenzyme subunits Skp1, Cul1 and Rbx1, and decreased ubiquitination activity. Loss of the LRRs also differentially affected Fbxl17 binding to its targets. Thus, genomic rearrangements in *FBXL17* are likely to disrupt SCF^Fbxl17^-regulated networks in cancer cells. To investigate the functional effect of these rearrangements, we performed a yeast two-hybrid screen to identify Fbxl17-interacting proteins. Among the 37 binding partners Uap1, an enzyme involved in *O*-GlcNAcylation of proteins was identified most frequently. We demonstrate that Fbxl17 binds to UAP1 directly and inhibits its phosphorylation, which we propose regulates UAP1 activity. Knockdown of Fbxl17 expression elevated *O*-GlcNAcylation in breast cancer cells, arguing for a functional role for Fbxl17 in this metabolic pathway.

**Electronic supplementary material:**

The online version of this article (10.1007/s00018-019-03306-y) contains supplementary material, which is available to authorized users.

## Introduction

The genomes of most common epithelial cancers, such as breast cancer, are highly rearranged, but our knowledge of the rearrangements and the genes they target remains rudimentary [1]. A few common, large-scale rearrangements have been known for some time, such as loss of the distal arm of 8p, 17p and 18q and the amplification of *ERBB2* in breast cancer, but many more less-frequently occurring aberrations remain to be characterised and may be diagnostically or therapeutically important. For example, the *EML4*-*ALK* fusion occurs in only approximately 5% of non-small cell lung cancers and is a target for therapy [2]. Genome sequencing has focused on point mutations in exomes, with only a few results for structural mutations reported so far, for limited sets of tumours [3–8]. Array-CGH detects larger scale unbalanced rearrangements and is available for large panels of tumours [9]. If such breaks fall within genes, they must at least inactivate that copy of the gene, and in some cases they will create truncated proteins or gene fusions. Some gene fusion data are also available, from genomic [3–8] or transcript sequencing [10]. From surveying these datasets, we determined that *FBXL17* is among the more frequently rearranged genes in a number of epithelial cancers, including breast, prostate and oesophageal cancers.

*FBXL17* encodes a little-studied member of the F-box family of proteins (FBPs). They are components of the ubiquitin conjugation pathway, which, by directing the ubiquitination of target proteins, regulate major cellular processes that require rapid alterations in protein levels, activity and localisation, such as cell cycle progression, cell signalling, and receptor recycling [11]. Ubiquitination of proteins requires an enzymatic cascade involving an E1 ubiquitin-activating enzyme, an E2 ubiquitin-conjugating enzyme, and an E3 ubiquitin ligase [12]. FBPs are subunits of the SCF (Skp1-Cul1-F-box protein)-type E3 ubiquitin ligases, which utilise protein–protein interaction domains, like leucine-rich or WD40 repeats, to recruit substrates to the ligase. FBPs bind an adaptor protein Skp1 through their F-box domains (FBD), and the FBP:Skp1 dimer is a switchable unit that docks with a cullin scaffold and Rbx1 (Ring finger domain containing protein), which in turn recruits a ubiquitin-charged E2 ligase. The Cand1 protein actively dissociates the pool of FBP:Skp1 dimers from cullin, regulating the levels of active E3 ligases in the cell [13, 14]. FBPs not engaged as part of active E3 ligases also have functions outside of the Ubiquitin Proteasome System (UPS) [15].

Several FBPs have oncogenic and/or tumour suppressive activities [16, 17]. Indeed, the first FBP described, Skp2 (S-phase kinase-associated protein 2, Fbxl1) is activated by amplification in several cancers, including breast, lymphoma, non-small cell lung cancer and glioblastoma [18, 19]. Skp2 is thought to have its main oncogenic activity by promoting the degradation of the cyclin-dependent kinase inhibitor, p27 [20–22]. However, loss of *SKP2* also induces senescence in response to oncogenic stimuli, such as Ras expression or the loss of *Pten* [23]. Fbxw7 is a tumour suppressor, inactivated in approximately 7% cancers [24], which targets the turnover of important oncogenes, such as Myc, cyclin E, and Notch [16]. β-TrCP (*BTRC*, Fbxw11) is also mutated in several cancers, including breast and colorectal cancer and melanoma, potentially stabilising its oncogenic substrate β-catenin [19]. The true extent of FBP dysregulation in cancer, particularly through genomic rearrangements, is unknown. We found *FBXL17* is rearranged in breast cancers, and these rearrangements often disrupt the LRRs of Fbxl17. Loss of LRRs leads to a differential loss of interaction with Fbxl17 binding partners, and prevents its assembly into a functional SCF complex. We show that Fbxl17 interacts with Uap1, UDP-*N*-acetylglucosamine pyrophosphorylase 1, to regulate the overall levels of* N*-acetylglucosamine modification (*O*-GlcNAcylation) of proteins in cells. Our data support a model whereby Fbxl17 has tumour suppressor activity in breast cancers.

## Results

### *FBXL17* is rearranged in cancer

**Fig. 1:**
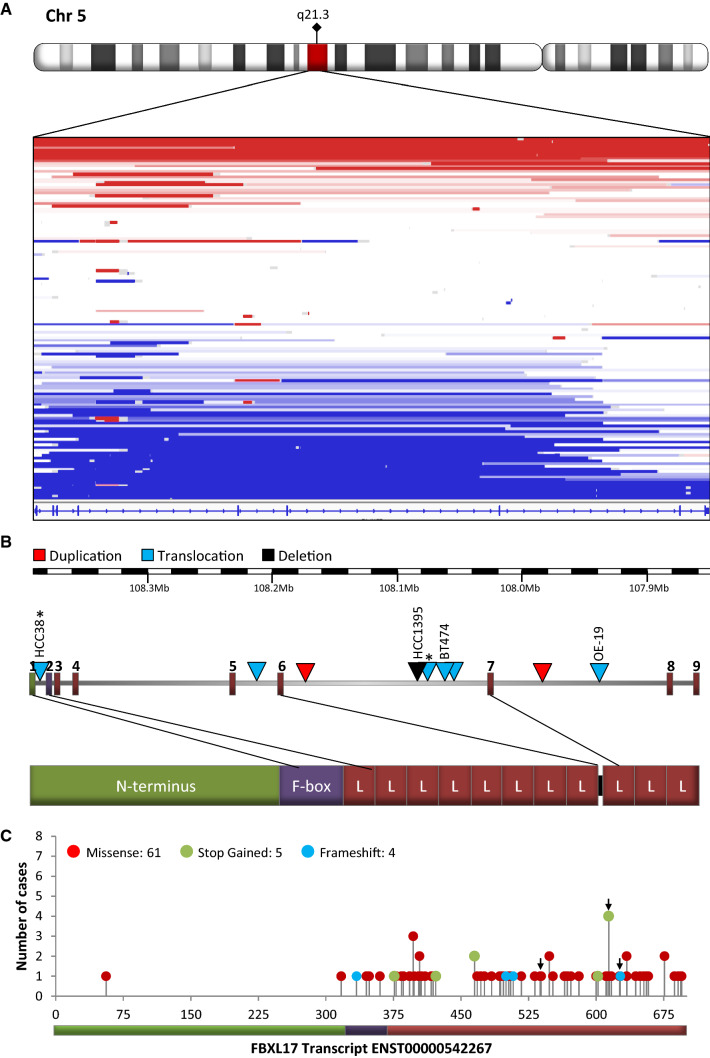
Breaks in *FBXL17* and the proteins encoded. **a** Breaks in 135/1992 breast tumours [9], detected as copy number steps by array-CGH. X-axis is genomic sequence of *FBXL17*, reversed since *FBXL17* is a negative strand gene. Each horizontal line represents a tumour, with breaks indicated by colour change. Blue, copy number loss; white, no change; red, gain. **b***FBXL17* exons from Ensembl transcript ENST00000542267.5 (Refseq NM_001163315.2, NP_001156787.2), chr5:107,859,045-108,382,098 in GRCh38/hg38. Triangles indicate breakpoints in *FBXL17* identified in cell lines (as labelled) or primary breast tumours (unlabelled), Asterisk indicates known fusion. Bottom, protein domains of Fbxl17 scaled to protein sequence. L, leucine-rich repeat. **c** Non-synonymous somatic mutations mapped to Fbxl17 as reported by The Cancer Genome Atlas (TCGA), arrows indicate breast cancer associated mutations. Schematic underneath represents Fbxl17 domains, green, N-terminus, purple, F-box domain, red, leucine-rich repeats

To identify genes that are rearranged in breast cancers, we scanned segmented array-CGH copy number data for 1992 primary breast tumours [9]. 135 (7%) had at least one genomic break within *FBXL17*, detected as a copy number step, distributed in various ways (Fig. 1a). The majority of these copy number losses or gains occurred at the 3′ end of *FBXL17*. Given the LRRs of Fbxl17 are encoded from exon 3 onwards it is likely these protein–protein interacting domains are disrupted by such rearrangements.

In addition to breaks in tumours, array-CGH data showed breaks in *FBXL17* in four cancer cell lines, the breast carcinoma cell lines, BT-474, HCC38, and HCC1395, and the oesophageal/gastric cardia adenocarcinoma line OE-19 [25]. The breaks were verified by FISH (Figs. 1b and S1). In BT-474, one of three copies of *FBXL17* was broken, with retention of the 3′ end, exons 7–9 (Figs. 1b and S1B). Both HCC38 and OE-19 had an extra copy of the 5′ end of *FBXL17*, up to intron 1 and intron 7, respectively (Figs. 1b and S1B). In HCC38, this break was confirmed to be the *FBXL17*-*PJA2* fusion transcript reported in [10] (Fig. S1C, S1F and S1G). In HCC1395, both array-CGH (Fig. S1H) and paired-end sequencing showed an internal homozygous deletion in *FBXL17* between exons 6 and 9 [5, 25] verified by RT-PCR and FISH (Fig. S1D, S1E and S1I) which would truncate Fbxl17 near its C-terminus and encode a mutant protein lacking approximately three LRRs (Fig. 1b).

Because the cell line examples may not be typical, we looked for examples of *FBXL17* rearrangements in breast cancers, in paired-end whole-genome DNA sequencing data from 250 primary breast tumours of the Cambridge Personalised Breast Cancer Programme. Rearrangements (‘Structural variants’) in *FBXL17* were identified in five of the tumours. Manual inspection of RNA sequences from these five tumours confirmed that two of the rearrangements were transcribed as predicted: a translocation joining exon 6 to an undocumented exon on chromosome 7 and a duplication of exon 6, respectively (Fig. 1b; Supplementary Table 1). A third case with a breakpoint in intron 6 showed unspliced transcription from exon 6 into intron 7; however, we cannot rule out that this was normal unspliced RNA. Serendipitously, a further RNA sample, inspected because it had a rearrangement which did not pass filtering, showed splicing from exon 6 into exon 4. This suggested the presence of a rearrangement which was not detected by DNA sequencing in an additional tumour. The partial agreement we find between RNA and DNA sequencing is expected as both methods lack sensitivity to identify all rearrangements [26]. Thus, consistent with the cell line rearrangements, the breakpoints in the tumours fell within introns that would disrupt the expression of LRRs, with the majority (4/6) occurring in intron 6 (Fig. 1b, Supplementary Table 1). There was no clear relationship between *FBXL17* rearrangement and any molecular classification [27] of the tumours and cell lines, although five of the six tumours and the breast cell lines were *TP53 *mutant (Supplementary Table 1). Of the tumours, three were oestrogen receptor (ER) positive, and three were ER-negative. One tumour and one cell line were *ERBB2/HER2* positive. We also classified these tumours into the 11 IntClust sets [27], and they fell into three sets: clusters 4, 7 and 10. In addition, classifying these six cases using PAM50 breast cancer subtyping, gave four basal and two luminal A cases. Although the number of cases is small, these data suggest Fbxl17 is not rearranged in a particular cancer subtype.

Rearrangements of *FBXL17* have also been detected in other epithelial cancers (Supplementary Table 2) including prostate [8] and oesophageal adenocarcinoma [7]. Many of these rearrangements are also predicted to truncate Fbxl17, resulting in loss of LRRs. TCGA (Cancer Genome Atlas project) data was mined for genomic alterations affecting *FBXL17* using cBioPortal (http://cbioportal.org) [28]. Perhaps most striking was the TCGA mapping of non-synonymous somatic *FBXL17* mutations. Mutations in *FBXL17* almost exclusively (68/70) target its C-terminus containing the FBD and LRRs (Fig. 1c).

In summary, *FBXL17* is broken in approximately 7% of breast cancers, and additionally rearranged or mutated in other epithelial cancers. At least some of the breaks truncate Fbxl17, removing some or all of the LRRs and sometimes also the FBD. Examples of truncation are present in three cancer cell lines, and rearrangements have been confirmed in primary breast tumours. These genomic alterations suggest the ability of Fbxl17 to recruit substrates for ubiquitination or to form part of an SCF complex may be compromised.

### Deletion of LRRs in Fbxl17 compromises ubiquitination activity due to impaired recruitment of SCF subunits

**Fig. 2:**
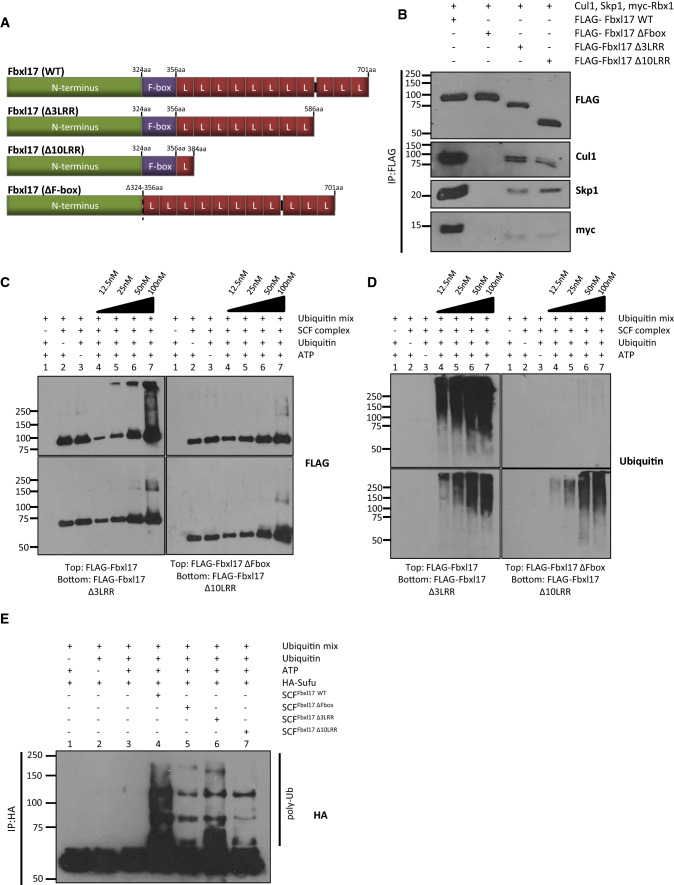
Loss of Fbxl17 LRRs impairs recruitment of SCF^Fbxl17^ subunits and SCF^Fbxl17^ ligase activity. **a** Schematic showing Fbxl17 constructs used to make SCF ligases, all contain an N-terminal FLAG tag (not shown). L, leucine-rich repeat. **b** A representative immunoblot for SCF holoenzyme components that co-immunoprecipitate with FLAG-Fbxl17 and mutant Fbxl17 constructs FLAG-Fbxl17ΔFbox, FLAG-Fbxl17Δ3LRR and Fbxl17Δ10LRR, *n* = 4. **c** Titration of the auto-ubiquitination activity of purified SCF^Fbxl17^ ligase complexes. A concentration gradient (12.5 nM, 25 nM, 50 nM, 100 nM) of purified SCF^Fbxl17^ or mutant complexes SCF^Fbxl17ΔFbox^, SCF^Fbxl17Δ3LRR^ or SCF^Fbxl17Δ10LRR^ was used in an in vitro ubiquitination assay in the presence of a ubiquitin mix (ubiquitin buffer, UBE1, UbcH5a and ATP). Following SDS-PAGE membranes were probed with anti-FLAG antibody to detect SCF^Fbxl17^ ligases, *n* = 2. **d** As (C) but probed with anti-ubiquitin antibody, *n* = 2. **e** In vitro ubiquitination assay of SCF^Fbxl17^ and mutant ligase complexes in combination with HA-tagged substrate Sufu in the presence of a ubiquitin mix as in **c**. Proteins resolved by SDS-PAGE and membrane probed with anti-HA antibody, *n* = 3

As most of the genomic rearrangements in *FBXL17* are predicted to target its LRRs, we wanted to investigate the effect of their loss on Fbxl17 ligase activity. We used co-immunoprecipitation assays to check the incorporation of Fbxl17 into an SCF E3 ligase. HEK293T cells were co-transfected with the subunits of SCF ligases, Skp1, Cullin1, Rbx1, and various N-terminally FLAG-tagged Fbxl17 constructs (full-length Fbxl17 (1-701aa), an internal FBD deletion, Fbxl17ΔFbox (Δ324-358aa) and two LRR-truncation constructs Fbxl17∆3LRR (1-586aa), and Fbxl17∆10LRR (1-384aa) (Fig. 2a). 48 h post transfection, cells were lysed, immunoprecipitated with FLAG antibodies, and blotted for the associated SCF subunits (Figs. 2b and S2B). While Skp1, Rbx1 and Cullin1 co-immunoprecipitated efficiently with WT Fbxl17, these components were reduced in the immunoprecipitates of the truncation mutants. For example, Cullin1 binding to Fbxl17Δ3LRR was reduced by 81% (*p *= 4.12E−04; n = 4) and Fbxl17Δ10LRR by 82% (*p *= 1.28E−06; *n* = 4) relative to WT Fbxl17, while Skp1 binding to Fbxl17Δ3LRR was reduced by 77% (*p *= 5.52E−05; *n* = 5) and Fbxl17Δ10LRR by 67% (*p *= 2.74E−05; *n* = 4) relative to WT Fbxl17, despite these truncations having intact FBDs (Figs. 2b and S2B). As expected, when the FBD was deleted in Fbxl17ΔFbox, none of the subunits were co-immunoprecipitated. These data indicate that in addition to the FBD, the LRRs of Fbxl17 facilitate the assembly of the SCF^Fbxl17^ ligase.

Since the Cullin1 and Rbx1 subunit allow E2 recruitment, these data suggest that the mutant SCF^Fbxl17^ ligases will have reduced activity. We tested this by performing in vitro ubiquitination assays of purified SCF complexes, assembled with either WT or LRR-truncated Fbxl17 proteins, in the presence of an E1 and E2 enzyme. We first tested the ability of the mutant Fbxl17 proteins to promote auto-ubiquitination as part of an SCF E3 ligase (Figs. 2c, d and S2A). We observed higher molecular weight bands using an antibody raised against Fbxl17 using 25 nM of SCF^Fbxl17^ in in vitro ubiquitination reactions, and the signal intensified with increasing concentrations of the WT ligase. In parallel assays, 50 nM of mutant SCF^Fbxl17∆3LRR^ ligase showed residual activity which increased at 100 nM, but it was considerably less than WT SCF^Fbxl17^ ligase. SCF^Fbxl17Δ10LRR^ ligase showed the greatest reduction in activity (Fig. 2c), comparable to the inactive SCF^Fbxl17ΔFbox^ mutant. The difference in activity of the SCF complexes was even more apparent when the membranes were probed for ubiquitin (Fig. 2d). SCF^Fbxl17ΔFbox^ had no ligase activity, while E3 ubiquitin ligases made with the LRR-truncated Fbxl17 mutants had reduced ligase activity compared to WT Fbxl17.

To test whether the ligases made by WT or mutant versions of Fbxl17 could ubiquitinate a heterologous substrate, we performed in vitro ubiquitination assays using Sufu (Fig. 2e) [29]. HA-Sufu was purified from HEK293T cells by immunoprecipitation. High molecular weight smears can be seen after the addition of SCF^Fbxl17^ (Fig. 2e, lane 4) and to a much lesser extent SCF^Fbxl17Δ3LRR^ (Fig. 2e, lane 6). Both SCF^Fbxl17ΔFbox^ and SCF^Fbxl17Δ10LRR^ show greatly reduced ubiquitination activity. Together these data indicate that the LRRs in Fbxl17 contribute to assembly of the SCF E3 ligase and its ligase activity.

### Fbxl17 interacting proteins identified by yeast-two hybrid screening

Our data suggest that if Fbxl17 is mutated in the LRR-encoding region, the proteins interacting with them will be mis-regulated as a result of aberrant SCF assembly and its effects on ligase activity. We performed a yeast two-hybrid screen to identify Fbxl17 interacting partners. To focus the screen on LRR-binding partners, we engineered the bait plasmid to contain the FBD and LRRs (321-701aa) of Fbxl17 but omitted its N-terminus. 37 unique prey, cloned in-frame to the Gal4 activation domain (GAD), were identified as candidate partners for Fbxl17 (Table 1; Fig. S3A). More than a third (13/37) of the prey were isolated independently at least twice. The most common prey plasmids isolated encoded GAD fusions to UDP-*N*-acetylglucosamine pyrophosphorylase 1 (Uap1) and to ubiquitin-fold modifier conjugating enzyme 1 (Ufc1), which were isolated 26 and 13 times, respectively. Moreover, since multiple, non-identical plasmids were isolated, a minimal common region defined a likely interacting domain within it, e.g. aa 357-505 at the C-terminus of Uap1 (Table 1). Additionally, Klhl12 and Klhl7, two members of the Kelch-like family of proteins, casein kinase 2b (Csnk2B), rearranged L-Myc fusion (Rlf), and C21orf91 were repeatedly isolated. To test whether the interaction between Fbxl17 and its prey was dependent on its LRRs, three LRRs (Δ3LRR) were deleted from the bait plasmid. Although this truncation did not affect Fbxl17 expression (Fig. S3B), none of the yeast co-transformed with the Δ3LRR bait plasmid and the various prey grew under the selective conditions requiring a bait-prey interaction. These results indicated Fbxl17 interaction with its prey was dependent on its three C-terminal LRRs (Fig. S3A).

**Table 1:** Fbxl17 interacting proteins

Gene symbol	Full name	isolates	Minimal region (aa)	Full-length size (aa)	Modified
*UAP1*	UDP-*N*-acetylglucosamine pyrophosphorylase 1	26	357–505	505	Ub
*UFC1*	Ubiquitin-fold modifier conjugating enzyme 1	13	12–95	167	Ub
*CSNK2B*	Casein kinase 2, beta polypeptide	4	1–123	215	Ub
*KLHL12*	Kelch-like 12 (Drosophila)	4	1–162	568	
*RLF*	Rearranged l-myc fusion	4	1670–1914	1914	
*C21orf91*	EURL/Chromosome 21 open reading frame 91	4	1–178	296	
*ACSBG2*	Acyl-CoA synthetase bubblegum family member 2	3	528–666	666	
*ETFA*	Electron-transfer-flavoprotein, alpha polypeptide	3	103–284	333	Ub
*METAP2*	Methionyl aminopeptidase 2	3	323–342	478	Ub
*AKD1*	Adenylate kinase domain containing 1	2	415–624	1911	
*SCG5*	Secretogranin V (7B2 protein)	2	1–188	212	
*TASP1*	Taspase, threonine aspartase, 1	2	135–318	420	
*KLHL7*	Kelch-like 7 (Drosophila)	2	37–240	586	Ub
*PHF7*	PHD finger protein 7	1	50–236	381	
*ZMYM2*	Zinc finger, MYM-type 2	1	141–390	1377	
*IDO1*	Indoleamine 2,3-dioxygenase 1	1	14–128	403	
*PSME4*	Proteasome (prosome, macropain) activator subunit 4	1	367–420	1843	Ub
*PPP3CB*	Protein phosphatase 3, catalytic subunit, beta isozyme	1	381–496	524	Ub
*ZNF350*	Zinc finger protein 350	1	310–532	532	
*TGFBI*	Transforming growth factor, beta-induced, 68 kDa	1	115–326	683	
*SCPEP1*	Serine carboxypeptidase 1	1	273–452	452	Ub
*FILIP1L*	Filamin A interacting protein 1-like	1	508–705	1135	
*ACPL2*	Acid phosphatase-like 2	1	266–464	480	
*TPP2*	Tripeptidyl peptidase II	1	879–1141	1249	Ub
*OLR1*	Oxidized low density lipoprotein (lectin-like) receptor 1	1	124–273	273	
*USP25*	Ubiquitin specific peptidase 25	1	323–450	1055	Ub
*FAM190A*	Family with sequence similarity 190, member A	1	555–726	900	
*PCCB*	Propionyl CoA carboxylase, beta polypeptide	1	172–468	539	
*TMOD1*	Tropomodulin 1	1	66–334	359	
*IFT46*	Intraflagellar transport 46 homolog (Chlamydomonas)	1	1–216	304	
*SRBD1*	S1 RNA binding domain 1	1	260–491	995	
*MED14*	Mediator complex subunit 14	1	1053–1282	1454	Ub
*CCDC147*	Coiled-coil domain containing 147	1	478–684	872	
*HADH*	Hydroxyacyl-CoA dehydrogenase	1	112–261	261	Ub
*CLPX*	ClpX caseinolytic peptidase × homolog (E. coli)	1	42–341	633	
*COG2*	Component of oligomeric golgi complex 2	1	457–534	738	Ub
*TMEM126A*	Transmembrane protein 126A	1	89–195	195	Ub

Minimal region denotes the amino acids present in all interacting cDNA clones; full-length size is the predicted size of the protein. Ub denotes protein is ubiquitinated in [39]

### Fbxl17 interacts with Uap1, Ufc1, Klhl12 and Csnk2B in human cells in vivo

**Fig. 3:**
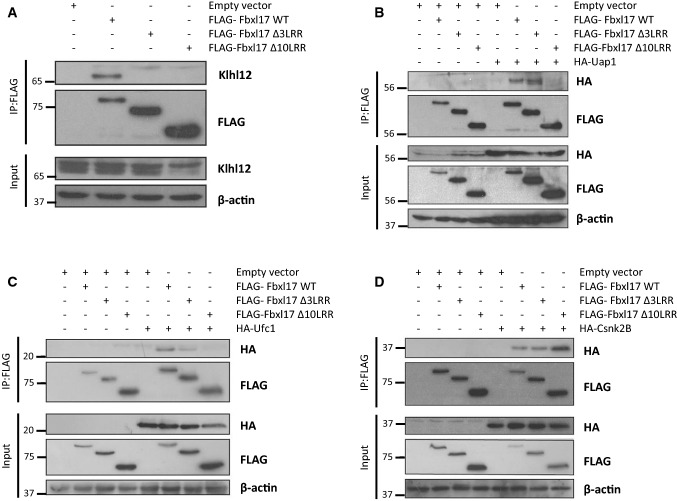
Deletion of the LRRs of Fbxl17 affects its binding to different proteins to different extents. **a** Immunoprecipitates; using anti-FLAG beads, of HEK293T cells expressing FLAG-Fbxl17, FLAG-Fbxl17∆3LRR or FLAG-Fbxl17∆10LRR, probed for endogenous Klhl12. **b–d** As (**a**), but with co-expression of exogenous HA-tagged Uap1 (**b**), Ufc1 (**c**), or Csnk2B (d) and probed with anti-HA antibody

To validate the yeast two-hybrid results, we tested Uap1, Ufc1, Csnk2B and Klhl12 for their interaction with Fbxl17 in human cells, using co-immunoprecipitation assays. All four proteins tested were detected in immunoprecipitates of FLAG-tagged Fbxl17 (Fig. 3a–d). We noted truncation of LRRs resulted in increased expression of mutant Fbxl17 (Fig. 2b–d). Despite their enhanced expression, Klhl12 did not interact with either the ∆3LRR or ∆10LRR mutants, indicating its interaction with Fbxl17 was dependent on the LRRs (Fig. 3a). Uap1 and Ufc1 both co-immunoprecipitated with WT and ∆3LRR Fbxl17, but truncation of 10 LRRs ablated their interaction (Fig. 3b, c). Uap1 was also present in immunoprecipitates of the mutant FLAG-Fbxl17∆Fbox indicating that Uap1 binding is dependent on LRR2-8 in Fbxl17 (Fig. S3C). In contrast to the other partner proteins, HA-Csnk2B co-immunoprecipitated roughly equivalently with WT, ∆3LRR, and ∆10LRR constructs, suggesting that their interaction does not rely on the LRRs and thus may interact via the FBD or N-terminus of Fbxl17 in human cells (Fig. 3d).

The diminished interactions of truncated Fbxl17 with some of its binding partners could be caused by a change in its subcellular distribution. Cellular fractionation and immunofluorescence assays were conducted in parallel to determine the distribution of the WT and mutant proteins in cells. Endogenous Fbxl17 was present in both cytoplasmic and nuclear fractions (Figs. S3D, S4A). Immunofluorescence showed all transfected Fbxl17 constructs showed a predominantly nuclear localisation and also weaker cytoplasmic staining (Fig. S4A). This argues against an altered localisation preventing the mutant forms from interacting with its binding partners.

In sum, these data validate results from the yeast two-hybrid screen since the binding partners identified also interact in human cells with the full length Fbxl17 protein. Furthermore, the association of Klhl12, Ufc1, and Uap1 with Fbxl17 was dependent on its LRRs, as truncation of this region weakened or ablated their interaction.

**Fig. 4:**
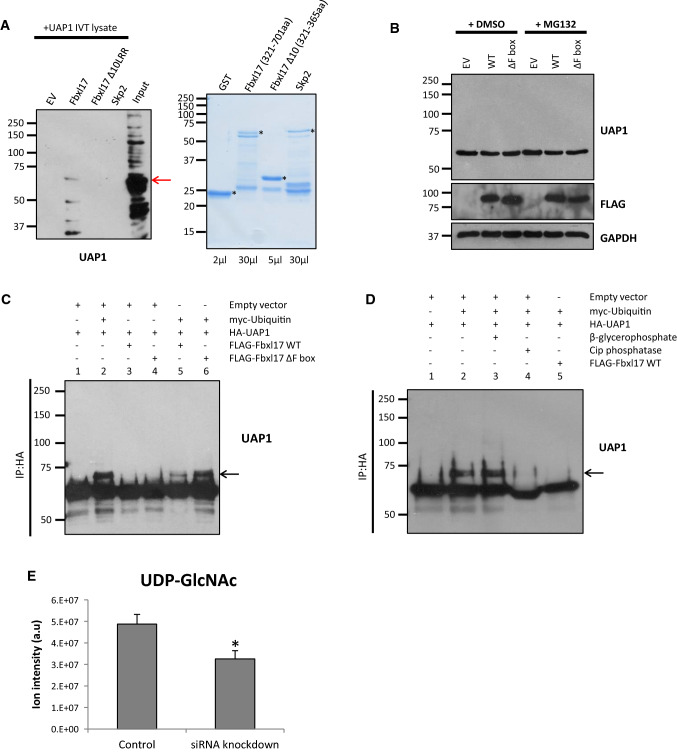
Fbxl17 inhibits the phosphorylation of UAP1. **a** In vitro GST pull-down assay using bacterially expressed and purified Fbxl17 constructs or Skp2/GST as controls immobilised on a GST column incubated with rabbit reticulocyte lysate (left panel). Fbxl17(321-701aa) and Skp2 constructs contained an IRES_Skp1 to aid expression. Input for rabbit reticulocyte lysate = 20%. Coomassie staining of GST proteins, volume of sample loaded indicated below lanes (right panel) * indicates bands relating to expressed proteins, arrow represents Uap1, *n* = 2. **b** HEK293T cells transfected with Fbxl17WT, ΔFbox or empty vector (EV) for 48 h then treated with 10 µM MG132 or DMSO for 4 h. Whole cell lysates immunoblotted with the indicated antibodies, *n* = 3. **c** In vivo ubiquitination assay for UAP1. HA-UAP1 immunoprecipitated from HEK293T cells transfected with ubiquitin and indicated Fbxl17 constructs. Membranes probed with anti-UAP1 antibody, arrow indicates modified Uap1, *n* = 3. **d** In vivo ubiquitination assay for UAP1 as in **c** in the presence of β-glycerophosphate (lane 3) and alkaline phosphatase (CIP) (lane 4), *n* = 2. **e** LC–MS analysis of total UDP-GlcNAc levels in U2OS cells treated with Fbxl17 siRNA3 or control siRNA for 48 h. Mean ± SEM for five biological replicates, *p < 0.05

To test the directness of the interaction between Fbxl17 and one of its interacting proteins, we performed a GST pull-down assay using Uap1. We tested GST-Fbxl17(321-701aa), GST-Fbxl17Δ10LRR(321-383aa), and GST-Skp2, another LRR-containing FBP, and GST only were used as controls. GST-FBP proteins were co-expressed with an IRES-Skp1 to facilitate expression in bacteria, with the exception of Fbxl17Δ10LRR, which was robustly expressed. GST-FBPs were immobilised on a GST column and incubated with in vitro transcribed and translated Uap1. Following binding assays, samples were resolved by SDS-PAGE, and membranes probed with antibodies to Uap1. We observed Uap1 binding specifically to GST-Fbxl17(321-701aa), but not to GST only or to GST-Skp2. Moreover, deletion of 10 LRRs abolished Uap1 binding to Fbxl17 (Fig. 4a). These results indicate Fbxl17 interacts directly with Uap1, and this occurs via its LRRs.

### Fbxl17 inhibits the phosphorylation of Uap1

To test the functional significance of Fbxl17 interaction with Uap1, we over-expressed Fbxl17 and monitored the steady state levels of Uap1 by immunoblotting. We found Uap1 levels were unchanged in the presence of MG132 or with increased levels of Fbxl17 (Fig. 4b), which suggests it does not promote the proteasomal degradation of Uap1. We next tested whether Uap1 was a substrate of SCF^Fbxl17^ ligase in vivo, by co-transfecting cells with HA-Uap1, Myc-ubiquitin, and FLAG-Fbxl17 (WT or ∆F-box domain) constructs. However, we found no evidence of laddering or smearing of Uap1, indicative of its poly-ubiquitination. Instead, we detected a discrete, higher molecular weight species of Uap1 upon transfection of Myc-ubiquitin (Fig. 4c, lane 2). Moreover, levels of this modified form of Uap1 were reduced when Fbxl17 was overexpressed (Fig. 4c, lane 5), indicating Fbxl17 opposed this modification of Uap1. Interestingly, this reduction of Uap1 modification was not observed when Fbxl17ΔFbox was overexpressed, (Fig. 4c, lane 6) suggesting this effect was dependent on Skp1 binding and/or the ligase activity of Fbxl17.

To determine the type of modification this higher molecular weight species of Uap1 represented, we immunoblotted with antibodies to ubiquitin and to Myc-epitope tag (Myc-ubiquitin). Surprisingly, these antibodies did not yield any signal in Uap1 immunoprecipitates, despite the overexpression of ubiquitin (S2C and S2D). These results indicated that the post-translational modification present on Uap1 was not ubiquitination. Based on the PhosphoSitePlus database, where multiple studies report Uap1 to be a phosphorylated protein, we tested whether this modified form of Uap1 represented a phosphorylated form. We conducted the in vivo ubiquitination assay in the presence of the phosphatase inhibitor β-glycerophosphate and alkaline phosphatase (CIP). Strikingly, the levels of modified Uap1 were almost completely absent following CIP treatment, suggesting the higher molecular weight species represented a phosphorylated form of Uap1 (Fig. 4d, lane 4). In sum, these data show that the overexpression of ubiquitin increases Uap1 phosphorylation, and Fbxl17 overexpression prevents this modification, and this function is dependent on its FBD.

### *FBXL17* knockdown results in increased levels of *O*-GlcNAcylation

Since Fbxl17 overexpression reduced the abundance of a phosphorylated Uap1, but not Uap1 steady state levels, we reasoned Fbxl17 might regulate Uap1 activity. Uap1 catalyses the formation of UDP-*N*-acetylglucosamine (UDP-GlcNAc), which is used by the glycosyltransferase *O*-GlcNAc Transferase (OGT) to add *N*-acetylglucosamine in *O*-glycosidic linkages to nuclear and cytosolic proteins. UAP1 is the main enzyme synthesizing UDP-GlcNAc [30–34]. Since our results indicate Fbxl17 opposes Uap1 phosphorylation, we tested whether reducing Fbxl17 expression would affect the amount of UDP-GlcNAc in the cell. U2OS cells were transfected with an siRNA targeting Fbxl17 (Fig. S4B), metabolites were extracted from these cells and UDP-GlcNAc levels were determined by mass spectrometry. We observed a 36% decrease (*p *= 0.013; *n* = 5) in total UDP-GlcNAc levels in Fbxl17 knockdown cells (Fig. 4e), which suggests that Fbxl17 promotes Uap1 activity.

**Fig. 5:**
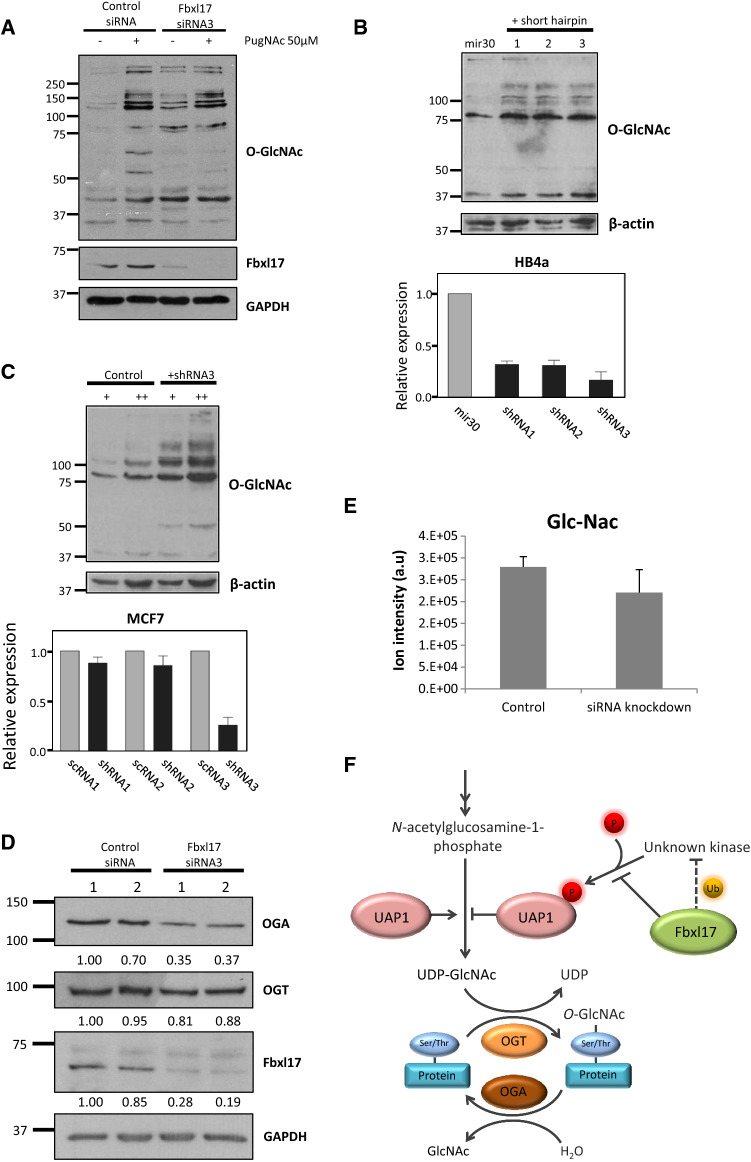
Knockdown of Fbxl17 increases total *O*-GlcNAcylation. **a** U2OS cells treated with Fbxl17 siRNA3 or control siRNA for 48 h followed by PugNAc treatment, 50 µM 3 h. Whole cell lysates immunoblotted with the indicated antibodies, *n* = 2. **b***FBXL17* mRNA knockdown by shRNA in HB4a immortalised normal breast cells (bottom panel). Expression normalised to GAPDH and plotted relative to miR30-infected control cells. Mean ± SEM of at least three independent experiments. *O*-GlcNAcylation monitored by immunoblotting with anti-*O*-GlcNAc antibodies (top panel). **c** FBXL17 mRNA knockdown by shRNA in MCF7 breast cancer cells (bottom panel). Expression normalised to scRNA-infected cells. Mean ± SEM of at least three independent experiments. Total *O*-GlcNAcylation monitored by immunoblotting with anti-*O*-GlcNAc antibodies. + and ++, 25 or 50 μg of protein lysate (top panel). **d** U2OS cells treated with Fbxl17 siRNA3 or control siRNA for 48 h. Whole cell lysates immunoblotted with the indicated antibodies. Band intensities quantified by densitometry and normalised to GAPDH expression (values below blots), *n* = 3. **e** LC–MS analysis of total GlcNAc levels in U2OS cells treated with Fbxl17 siRNA3 or control siRNA for 48 h. Mean ± SEM for five biological replicates. **f** Model of interaction between Fbxl17 and Uap1

To determine the downstream effects of Fbxl17 on this pathway, we next tested the effect of reduced Fbxl17 expression on the levels of *O*-GlcNAc modified cellular proteins. U2OS cells were treated with Fbxl17 siRNA as above, and cell lysates were immunoblotted for *O*-GlcNAc. Although UDP-GlcNAc levels were reduced in Fbxl17 KD cells (Fig. 4e), we observed increased levels of *O*-GlcNAc-modified proteins in Fbxl17 knockdown cells (Fig. 5a). This was also shown in two breast cell lines, HB4a and MCF7, by expression of shRNA constructs targeting *FBXL17* expression (Figs. 5b, c; S4C). This increase in *O*-GlcNAc modified proteins, despite reduced levels of UDP-GlcNAc, may be a result of changes in the expression or activity of the enzymes responsible for adding or removing *O*-GlcNAc, namely Ogt and Oga, which act downstream of Uap1 and UDP-GlcNAc. We therefore determined whether Ogt and Oga were changed following knockdown of Fbxl17. Although Ogt levels were unchanged, Oga levels were reduced which suggests the increased *O*-GlcNAcylated proteins is due to decreased Oga levels (Fig. 5d). Consistent with reduced Oga levels, mass spectrometry analysis of levels of GlcNAc, the product of Oga-mediated cleavage of *O*-GlcNAc modifications from proteins, were reduced by 18%, although this was not significant, in cells treated with Fbxl17 siRNA (Fig. 5e). Finally, we surveyed the expression of Fbxl17 and Uap1 expression in breast cancer datasets using the R2 platform (http://www.r2.amc.nl). Kaplan–Meier analysis for these genes revealed that breast tumours with either low Fbxl17 expression, or high Uap1 expression were associated with poorer survival in patients (Fig. S5).

## Discussion

Our results collectively indicate that *FBXL17* is frequently mutated in epithelial cancers in the genomic regions encoding its LRRs. We found *FBXL17* was rearranged in around 7% of breast cancers according to array-CGH, and also in cancer cell lines. Independently, analysis of sequence-level mutation data also suggested that *FBXL17* behaves like a tumour suppressor gene [35]. Many point mutations and breaks in *FBXL17* occurred in regions encoding its LRRs. These motifs are proposed to be the substrate docking sites within FBXL proteins, and are predicted to cause a failure to recruit substrates. Indeed, we find that progressive deletion of its LRRs caused decreased auto-ubiquitination and ubiquitination of a substrate, Sufu, by SCF^Fbxl17^. However, we also discovered that deleting LRRs impaired the assembly of the E3 ubiquitin-ligase complex. Both of the Fbxl17 LRR truncation mutants, ∆3LRR and ∆10LRR, bound Skp1 less well compared to WT, despite them containing the Skp1 binding motif. Reduced interactions with Cul1 and Rbx1 are likely due to less Skp1 binding, since FBPs do not interact directly with Cul1 or Rbx1 [36]. The C-terminus of an FBP has been shown in specific cases to contact Skp1 to stabilise the ligase [36], and our data show that truncating the LRRs of Fbxl17 also destabilises the SCF ligase. We predict that even if a truncated Fbxl17 were able to recruit some of its substrates, it would be less efficient in ubiquitinating them. Thus in the context of breast cancer, the rearrangements that target the LRRs of *FBXL17* would likely diminish ubiquitination of the network of SCF^Fbxl17^ substrates.

To investigate functional consequences of these rearrangements, we screened for proteins interacting with the LRR of Fbxl17. Our yeast two-hybrid screen identified 37 novel interactions, which was found to only minimally overlap with previous studies reporting Fbxl17 interacting proteins [37, 38]. Only eight proteins, Klhl12, Klhl7, Zmym2, Hadh, Clpx, Ppp3cb, Pccb, Srbd1 also appeared in these other studies. However, Uap1 and Ufc1, the most frequently recovered cDNAs in our screen, were not identified by either. The varied findings by different screening methodologies, indicates they identify distinct partner proteins and argues for a variety of experimental approaches to discovering protein interaction networks. Screens for interacting partners may identify substrates or regulators of FBPs. When validating four of the most repeatedly isolated prey cDNAs in mammalian cells, we noted differential binding (Csnk2B > Uap1 > Ufc1 > Klhl12) depending on the deletion of 3 or 10 LRRs. Notably, the Casein Kinase 2 subunit, Csnk2B, remained bound to Fbxl17 after deletion of 10 LRRs, suggesting that it binds to another region of Fbxl17, or other E3 ubiquitin ligase components, and may be a regulator of the ligase. Among the other interactors, Fbxl17 binding to Klhl12 or Ufc1 was decreased when the last three LRRs were truncated. However, Uap1 binding was disrupted only when ten LRRs were truncated suggesting Uap1 can bind to LRR2-8. These data led us to a model whereby the number of LRRs in Fbxl17, as dictated by the position of a rearrangement within *FBXL17,* would influence its interaction with its repertoire of partners. However, due to inefficient ligase assembly by LRR-truncated Fbxl17 mutants, we predict the ubiquitination of these proteins would be diminished. We note that 15 of the 37 proteins identified in the yeast two-hybrid screen are listed as being ubiquitinated proteins in a whole proteomic analysis of HEK293 cells (Table 1) [39].

There are not yet enough data on rearrangement of *FBXL17* in cancers to conclusively identify *FBXL17* inactivation as a driver mutation in cancer. Nonetheless, we were able to show a striking effect of reducing Fbxl17 expression on at least one important cancer-relevant pathway, in a relevant cell type, suggesting that inactivation of Fbxl17 would have a major effect on the cancer cell. This was through its regulation of Uap1, which is expressed in many breast cancers (Figs. S4D, S5D) and other cancer types [34, 40]. Surprisingly, we did not find Uap1 to be ubiquitinated by Fbxl17, but instead our results showed that increased Fbxl17 expression prevented the phosphorylation of Uap1. In addition, this inhibition of Uap1 phosphorylation was dependent on the Skp1-binding domain of Fbxl17, suggesting that SCF^Fbxl17^ ligase activity is essential. In contrast to Ogt and Oga [41], little is known about the regulation of Uap1, and our data suggest Fbxl17 positively regulates its activity (Fig. 5f). UDP-GlcNAc levels are significantly decreased when Fbxl17 is knocked down suggesting reduced Uap1 activity which is consistent with previous studies showing Uap1 expression is important for UDP-GlcNAc levels [34]. Although we have not identified the kinase responsible for phosphorylating Uap1, one possibility is that Fbxl17 ubiquitinates this kinase to inhibit its phosphorylation of Uap1 and promote Uap1 activity. Alternatively, Fbxl17 could shield Uap1 from this kinase, via a direct interaction between Fbxl17 and Uap1 (Figs. 4a, 5f).

We have shown that Fbxl17 regulates the *O*-GlcNAcylation pathway since reducing *FBXL17* expression in three cell lines increased global levels of *O*-GlcNAc-modified proteins. We did not assess glycosylation in the ER or Golgi, so cannot rule out a specific role for Fbxl17 there. However, the increase in global *O*-GlcNAcylation may be the result of greater utilisation of UDP-GlcNAc by Ogt, which would explain the lower UDP-GlcNAc levels and higher *O*-GlcNAcylation we observed in Fbxl17 knockdown cells. Although Ogt expression levels were unchanged, we cannot rule out that its activity is increased when Fbxl17 levels are reduced. However, the higher levels of *O*-GlcNAc modified proteins are likely due to the observed decrease in Oga expression. It has been proposed that there is an optimal level of global *O*-GlcNAcylation levels for cells to function and this is maintained by mutual regulation and balance of Ogt/Oga expression and activity [41, 42]. The decrease in Oga expression may represent a compensatory mechanism adopted by the cell to counteract the decrease in Uap1 activity and UDP-GlcNAc levels. *O*-GlcNAcylation is an important post-translational modification on many intracellular proteins—including p53, RNA polymerase II, the polycomb complex and Phosphofructokinase 1 (Pfk1), the main regulator of glycolysis—and is essential for viability of several mammalian cell types [43, 44]. Moreover, there is already considerable evidence that GlcNAcylation is altered in breast cancer and other cancers [45–48]. Caldwell et al. [49] found that breast cancer cells had increased *O*-GlcNAcylation and elevated OGT. Knocking down *OGT* inhibited tumour growth, decreased cell cycle progression, increased expression of the cell cycle inhibitor p27Kip1, and decreased invasiveness [49]. High nuclear and cytoplasmic O-GlcNAc was also observed in breast cancer patients with increased relapse rates, increased sites of distant metastases and poor outcome [50]. In breast cancer, low OGA levels are linked to higher grade tumours and metastasis [51]. We have shown that the regulation of Uap1 by Fbxl17 and an unidentified kinase, are factors in determining the levels of the *O*-GlcNAcylated proteome.

In conclusion, by surveying structural rearrangements in cancer databases, we discovered rearrangements commonly occur in *FBXL17* which affect its ability to bind substrates and also assemble as part of a functional SCF ubiquitin ligase complex. By screening for Fbxl17 interacting proteins, we discovered Uap1 as a binding partner, but not a substrate of Fbxl17, and established that Fbxl17 is a negative regulator of global O-linked GlcNAcylation. The loss-of-function mutations in *FBXL17* caused by structural rearrangements could have additional effects on the cell, since the targets of Fbxl17 are involved in major, cancer-relevant cellular processes.

## Materials and methods

### Yeast two-hybrid assay

The matchmaker gold yeast two-hybrid system (Clontech) was used to screen a human cDNA library (Mate & Plate™ Library—Normalized, Universal Human (Clontech)). Fbxl17 aa 321 to 701 was PCR amplified and subcloned into pGBKT7 (Clontech). aa 321 to 586 was amplified from pGBKT7-FBXL17 plasmid and subcloned into pGBKT7 to create the (∆3LRR) bait construct.

### Purification of SCF^Fbxl17^ complexes

HEK293T cells were transfected with SCF components (Skp1, Cul1, Myc-Rbx1) and FLAG-Fbxl17 constructs. After 48 h, cells were resuspended in lysis buffer (LB) (25 mM Tris–HCl, pH 7.5, 225 mM KCl, 1% NP-40) with a protease inhibitor cocktail (Sigma-Aldrich) and phosphatase inhibitors (10 mM NaF, 1 mM PMSF, 1 mM Na_3_VO_4_). Lysates were incubated with Anti-FLAG^®^ M2 Affinity Gel (Sigma-Aldrich) for 5 h at 4 °C with rotation. Beads were washed in LB and eluted in 300 µg/mL FLAG peptide (Sigma-Aldrich) in elution buffer (10 mM HEPES, pH 7.9, 225 mM KCl, 1.5 mM MgCl_2_, 0.1% NP-40) for 1 h at 4 °C with rotation. Purified SCF complexes were stored in 15% glycerol.

### In vitro ubiquitination assays

A screen of 10 different E2 enzymes determined that E2 UbcH5a enabled the most specific SCF^Fbxl17^ activity and was used in subsequent experiments. Purified SCF complexes at 12.5, 25, 50 and 100 nM were tested in the presence of a ubiquitin-mix (ubiquitin buffer, ubiquitin (20 µM) E1 (UBE1, 100 nM), E2 (UbcH5a, 500 nM) and Mg-ATP (2 mM) (Boston Biochem)) incubated at 30 °C for 90 min to determine ligase activity by auto ubiquitination. 50 nM of the SCF was sufficient for ligase activity and used in subsequent experiments. To test substrate ubiquitination substrates were transfected into HEK293T cells and immunoprecipitated using their indicated epitope tags conjugated to agarose beads. The purified substrate was then eluted from the beads and added as a component of the ubiquitin-mix. HA-Sufu was kindly provided by Vincenzo D’Angiolella (CRUK/MRC Oxford Institute for Radiation Oncology, Oxford, UK). Ubiquitination was detected by probing for the substrate or HA tag.

### In vivo ubiquitination assays

HEK293T cells were transfected with expression constructs of interest, including myc-ubiquitin, and treated with 10 µM MG132 (Sigma-Aldrich) 5 h prior to lysis. UAP1 was then immunoprecipitated with Monoclonal Anti-HA-Agarose antibody (Sigma-Aldrich). Modified UAP1 was detected with an endogenous UAP1 antibody. To test for phosphorylation 10 mM β-glycerophosphate or alkaline phosphatase (CIP) was added to the LB where indicated. LB containing CIP did not contain phosphatase inhibitors (NaF, Na_3_VO_4_).

### DNA constructs

Coordinates and exon numbers for the *FBXL17* gene are from Ensembl transcript ENST00000542267.5 (Fig. 1). Human *FBXL17* cDNA (GenScript) was subcloned into pcDNA3 and pcDNA3-FLAG. Truncation (∆3LRR) and (∆10LRR) and deletion constructs (ΔFbox) were constructed by amplification or two-step PCR mutagenesis. Human Ufc1 and Uap1 cDNAs were obtained from GeneArt. pcDNA3.1-FLAG-hKLHL12 was kindly provided by S. Angers (University of Toronto, Canada). pCK2_V2N1_Venus2-HA-CSNK2B_N1 was kindly provided by A. Beck-Sickinger (Leipzig University, Germany).

### Antibodies

The following antibodies were purchased anti-β-actin (Abcam, ab8227), anti-Cul1 (Santa Cruz, sc-11384), anti-Fbxl17 (Genetex, GTX119211), anti-FLAG^®^ M2 (Sigma-Aldrich, F3165), anti-Gal4 DBD (Santa Cruz, sc-510), anti-GAPDH (Sigma, G9545), anti-HA (Abcam, ab9110), anti-HA (C29F4) (Cell Signalling, 3724S), anti-Histone H1 (Santa Cruz, sc-8030), anti-Klhl12 (Abcam, ab14233), anti-myc tag (Cell Signalling, 2272), anti-*O*-GlcNAc (Covance, MMS-248R), anti-p19 (Skp1) (BD Biosciences, 610530), anti-UAP1 (Abcam, ab95949), anti-Ub (Santa Cruz, sc-8017), HRP-conjugated antibodies to mouse or rabbit IgG (Santa Cruz Biotechnology, sc-2055, sc-2313) or chicken IgY (Abcam, ab97135), Donkey anti-Rabbit and anti-Mouse IgG conjugated to Alexa Fluor 488 (Invitrogen). Signal detection was by enhanced chemiluminescence (ECL) (GE Healthcare).

### Cell culture, plasmids and transfection

HB4a is an immortalised normal breast epithelial cell line from M.J. O’Hare [52]. Cell lines were maintained in DMEM supplemented with 10% foetal bovine serum (ThermoFisher), 2 mM glutamine, 100 U/mL penicillin and streptomycin at 37 °C in a humidified 5% CO_2_ atmosphere. Where indicated, cells were treated with (50 µM) PugNAc, an inhibitor of *O*-GlcNAc-β-*N*-acetylglucosaminidase (Oga), for 3 h prior to cell lysis.

### Immunoblotting and immunoprecipitation analysis

Cells were lysed in protein extraction buffer (20 mM Tris–HCl pH 7.4, 1% IGEPAL, 1% Triton X-100, 50 mM NaCl, 2 mM EDTA pH 8, 30 mM NaP_2_O_7_ and protease inhibitor cocktail (Roche)). For the analysis of *O*-GlcNAcylated proteins, cells were lysed in RIPA buffer plus protease inhibitors.

For immunoprecipitation (IP) experiments, cells were lysed in hypotonic lysis buffer (10 mM Tris–HCl pH 7.5, 10 mM NaCl, 2 mM EDTA, 0.5% Triton X-100 and protease inhibitors) and immunoprecipitated with agarose-anti-HA (Sigma) or agarose-anti-FLAG (Anti-FLAG^®^ M2 Affinity Gel, Sigma-Aldrich) for 3.5 h. Beads were pelleted and washed four times in 1 × NET2 wash buffer (50 mM Tris–HCl pH7.5, 150 mM NaCl, 0.05% Triton X-100). Bound proteins were eluted by addition of 40 µL 2 × Laemmli sample buffer and incubation at 60 °C for 3 min.

### siRNA and shRNA expression

siRNAs were purchased from Eurofins genomics and transfected at a final concentration of ~ 60 nM using Lipofectamine™ RNAiMAX (ThermoFisher). Sequences for the siRNAs were as follows:

- siRNA2: GCAGAGAACTCAAAGATAT
- siRNA3: GGACAAACTCACTGATGAA

4 × 10^6^ ψNx cells were calcium phosphate transfected with shRNA or scRNA retroviral expression constructs with 25 μM chloroquine (Sigma-Aldrich). 2 days post-transfection, 2 × 10^6^ of target cells were infected with retroviruses in the presence of polybrene (Sigma-Aldrich).

Sequences for the shRNAs were as follows:

- shRNA 1: GGACAAACTCACTGATGAAGG (targets exon 3 of *FBXL17*);
- shRNA 2: GCTTGGACCTACGTCATATCA (targets exon 6 of *FBXL17*);
- shRNA 3: AGGCATGATCGTCATAGCTAA (targets exon 4 of *FBXL17*).

The following day, cells were selected using 1.7 μg/mL of puromycin. After selection, RNA was extracted, reverse transcribed and quantified by qPCR. Expression level was normalised to *GAPDH* expression and was plotted relative to the expression of *FBXL17* in the relevant control. Values represent mean ± SEM of at least three independent experiments.

### Metabolites extraction and LC–MS analysis

Cells were washed three times with PBS prior the extraction and 1 ml of extraction buffer (50% LC–MS grade methanol and 30% acetonitrile, 20% ultrapure water) was added per 1 × 10^6^ cells. Cell were then incubated on dry ice for 15 min, collected, kept under vigorous shaking for 15 min at 4 °C, and left for 1 h incubation at 20 °C. Samples were centrifuged at 13,000 rpm and supernatants were transferred to autosampler vials for LC–MS analysis. To avoid bias due to machine drift and processed blindly, samples were randomized. Q Exactive mass spectrometer (Thermo Fisher Scientific) coupled to a Dionex U3000 UHPLC (Thermo Fisher Scientific) system was used to perform the LC–MS analysis. Sequant ZIC-pHILIC column (150mm 3 2.1 mm) and guard column (20 mm 3 2.1 mm) (Merck Millipore) were utilized for the chromatographic separation and the column oven temperature was maintained at 40 °C. The mobile phase was composed of 20 mM ammonium carbonate and 0.1% ammonium hydroxide in water (solvent A), and acetonitrile (solvent B). The flow rate was set at 200 mL/min with the gradient was programmed as follows: initially stayed at 20% of A and 80% of B for 2 min, then subjected to a linear increase to 80% of A and decrease to 20% of B in 15 min. Both solvents were then brought back to initial condition and staid for 8 min. The mass spectrometer was operated in full MS and tSIM (targeted Single Ion Monitoring), in positive and negative mode. XCalibur Qual Browser and XCalibur Quan Browser software (Thermo Fisher Scientific) were used to acquire the spectra and analyse the data.

### Quantitative PCR

All qPCR reactions were performed in triplicates using LightCycler^®^ 480 SYBR Green Master Mix (Roche) according to manufacturer’s instructions. The relative expression ratio of a target gene in comparison to a reference gene in a cDNA panel was quantified [53]. GAPDH was used as an endogenous housekeeping transcript. The relative expression level was based on the difference in Ct values between a control cell line such as HB4a and a sample cell line in the cDNA panel.

### Electronic supplementary material

Below is the link to the electronic supplementary material.

Supplementary material 1 (DOCX 13 kb)

Supplementary material 2 (XLSX 16 kb)

Supplementary material 3 (PDF 1500 kb)
