# Supplementary material for: Fbxl17 is rearranged in breast cancer and loss of its activity leads to increased global O-GlcNAcylation
Source: Cell Mol Life Sci. 2019 Sep 27;77(13):2605–20. doi: 10.1007/s00018-019-03306-y (PMC7320043; doi:10.1007/s00018-019-03306-y)
Supplement: Supplementary file 1 — Supplementary material 1 (DOCX 13 kb) [file 18_2019_3306_MOESM1_ESM.docx]

**Supplementary Methods:**

**Breast cancer genomics data.** Copy number analysis from the METABRIC study: to remove polymorphic copy number variants, each tumour was compared to its matched normal, where available, or a pool of normal controls (1). There were no known variants in this interval.

Whole genome and transcriptome sequencing: data is from the first 250 cases of the Cambridge Personalised Breast Cancer Programme, led by JEA and CC, to be described elsewhere. DNA and RNA from consecutive consented patients’ tumour and matched blood were sequenced in paired-end mode by Illumina (Granta Park, Cambridge, UK). DNA sequencing coverage was tumour >110X, normal >35X; reads were 250bp. RNA was extracted using paired-end TruSeq Stranded Total RNA preparation kit (Illumina) and sequenced to a minimum coverage of 94 million pairs of 75bp reads. Structural variants and copy number aberrations were called by Illumina using Manta (2) following alignment with Isaac (3) to GRCh38 with decoy sequences. Structural variants were further filtered to remove calls with any supporting reads in the matched normal; calls also found in the pooled matched blood normal samples; and calls involving unassembled or mitochondrial chromosomes. RNA sequences were aligned with Bwa-mem (4) and inspected manually.

**Fluorescence *In Situ* Hybridisation**. Metaphase spreads and FISH were carried as described (5).  Chromosome 5 paint was amplified from chromosomes generously provided by Prof M. Ferguson-Smith.  BAC probes and fosmids were obtained from BACPAC Resources and checked by hybridization to normal metaphase chromosomes.

**Yeast methods**: Yeast protein extracts were prepared using the trichloroacetic acid (TCA) protein extraction method according to the Yeast Protocols Handbook (Clontech).

**Immunofluorescence.** U2OS cells were seeded onto glass coverslips and transfected 24h later with FLAG-Fbxl17 constructs. 48h post-transfection cells were washed in ice cold PBS, fixed in 4% PFA (in PBS) RT for 10min, permeabilised in 0.1% Triton X-100 (in PBS) RT for 10min and blocked in 0.2% fish skin gelatine (in PBS) for 30min. Endogenous Fbxl17 was immunostained with anti-Fbxl17 antibody and exogenous constructs with anti-FLAG antibody, both diluted in 0.2% fish skin gelatine. After 1h cells were stained with Alexa Fluor 488 conjugated secondary antibodies. Coverslips were then washed in PBS-T and ddH_2_O and mounted using 50% glycerol (in PBS) containing 1µg/ml DAPI counterstain. Images were collected using a Zeiss Confocal LSM700 Laser Scanning Microscope and ZEN imaging software.

**Cell fractionation.** 3 x 10^6^ cells were resuspended in 75µl Buffer A (10mM Hepes pH7.9, 10mM KCl, 1.5mM MgCl_2_, 0.34M Sucrose, 10% Glycerol, 1mM DTT, 0.1mM PMSF, protease inhibitors). Triton X-100 added to a final concentration of 0.1%, incubated on ice 5min and centrifuged 4 min 3,500 rpm 4°C. Supernatant removed (cytoplasmic fraction) and clarified by centrifugation, 15 min, 13,000 rpm, 4°C. Nuclear pellet washed in Buffer A + Triton and lysed in Diehl Buffer with protease inhibitors, incubated on ice 5 min and centrifuged 13,000 rpm 15 min, 4°C (nuclear fraction).

Supplementary Reference List

1. Curtis, C., et al., *The genomic and transcriptomic architecture of 2,000 breast tumours reveals novel subgroups.* Nature, 2012. **486**(7403): p. 346-52.

2. Chen, X., et al., *Manta: rapid detection of structural variants and indels for germline and cancer sequencing applications.* Bioinformatics, 2016. **32**(8): p. 1220-2.

3. Raczy, C., et al., *Isaac: ultra-fast whole-genome secondary analysis on Illumina sequencing platforms.* Bioinformatics, 2013. **29**(16): p. 2041-3.

4. Li, H. and R. Durbin, *Fast and accurate long-read alignment with Burrows-Wheeler transform.* Bioinformatics, 2010. **26**(5): p. 589-95.

5. Pole, J.C., et al., *High-resolution analysis of chromosome rearrangements on 8p in breast, colon and pancreatic cancer reveals a complex pattern of loss, gain and translocation.* Oncogene, 2006. **25**(41): p. 5693-706.
