# Supplementary material for: Fbxl17 is rearranged in breast cancer and loss of its activity leads to increased global O-GlcNAcylation
Source: Cell Mol Life Sci. 2019 Sep 27;77(13):2605–20. doi: 10.1007/s00018-019-03306-y (PMC7320043; doi:10.1007/s00018-019-03306-y)
Supplement: Supplementary file 3 — Supplementary material 3 (PDF 1500 kb) [file 18_2019_3306_MOESM3_ESM.pdf]

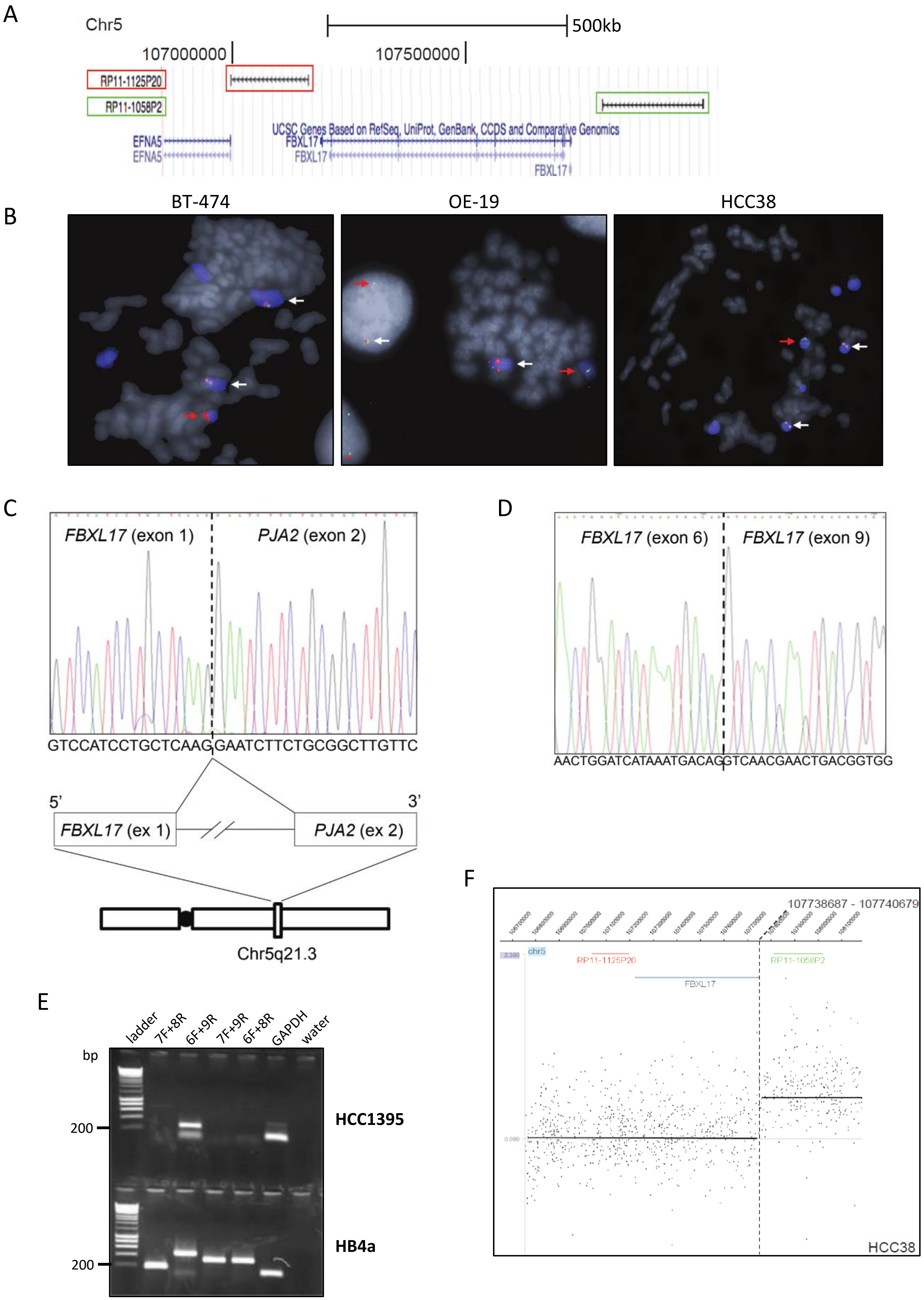
A
Chr5
500kb
107000000
107500000
RP11-1125P20
RP11-1058P2
UCSC Genes Based on RefSeq, UniProt, GenBank, CCDS and Comparative Genomics
EFNA5
EFNA5
FBXL17
FBXL17
FBXL17
B
BT-474
OE-19
HCC38
C
FBXL17 (exon 1)
PJA2 (exon 2)
GTCCATCCTGCTCAAGGAATCTTCTGCGGCTTGTTC
5'
3'
FBXL17 (ex 1)
PJA2 (ex 2)
Chr5q21.3
D
FBXL17 (exon 6)
FBXL17 (exon 9)
AACTGGATCATAAATGACAGGTCAACGAACTGACGGTGG
E
ladder
7F+8R
6F+9R
7F+9R
6F+8R
GAPDH
water
bp
200
200
HCC1395
HB4a
F
107738687 - 107740679
chr5
RP11-1125P20
RP11-1058P2
FBXL17
HCC38

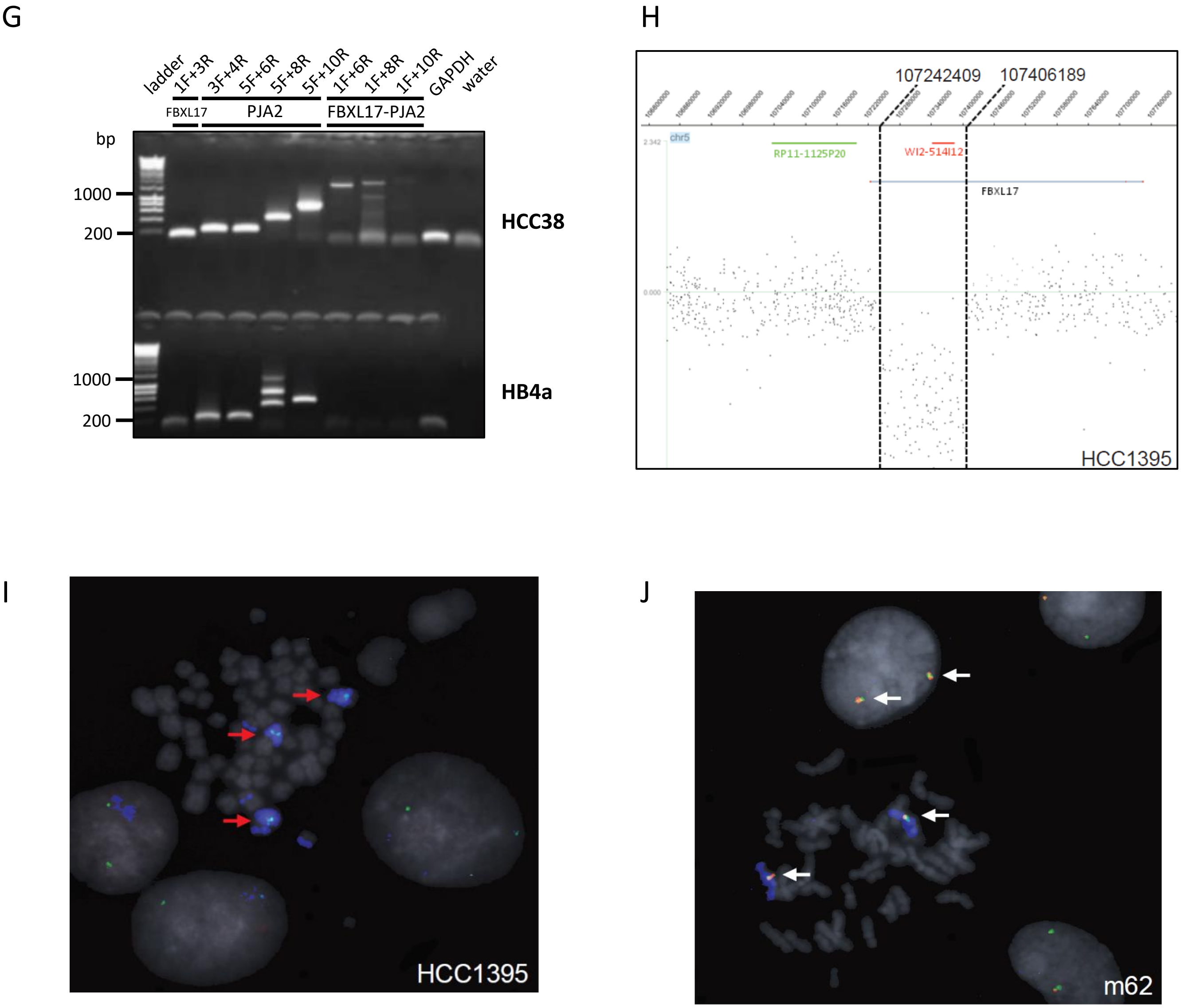

Supplementary Figure 1. Rearrangements of FBXL17 in breast cancer cell lines BT474, HCC38 and HCC1395, and oesophageal cancer cell line OE-19. (A) Schematic of FBXL17 with positions of the BACs (bacterial artificial chromosomes) used in FISH (reference genome hg19). **(B)** Breakpoint mapping by FISH: green, BAC flanking transcription start of the gene; red, BAC flanking end of the gene; blue, chromosome 5. White arrows, normal copies of FBXL17; red arrows, broken copies, identified by split signals. **(C)** cDNA sequence through junction between exon 1 of FBXL17 and exon 2 of PJA2 in HCC38. **(D)** cDNA sequence showing that exon 6 is spliced directly to exon 9 in HCC1395. **(E)** Absence of normal splicing between exons 6,7 and 8 in HCC1395, compared to normal breast line HB4a: RT-PCR using primers in exons 6,7,8 and 9 in various combinations. PCR products were sequenced. **(F)** Copy number of FBXL17 in HCC38, with location of BACs used in FISH mapping (hg18). **(G)** RT-PCR on HCC38 and normal breast line HB4a using primers in various combinations detects normal FBXL17, normal PJA2, and the FBXL17-PJA2 fusion transcript. PCR products were sequenced. **(H-J)** Homozygous deletion in HCC1395. **(H)** Copy number of FBXL17 region. Dashed lines, breakpoints in FBXL17. Approximate genomic position (hg18) indicated above. **(I,J)** FISH. Green, BAC RP11-1125P20 flanking end of FBXL17; red, fosmid WI2-514I12 (G248P8998E6) chr5: 107,314,720 – 107,356,686 (hg19) from the deleted interval, as shown in C. **(I)** FISH: all three copies lack fosmid signals from the deleted interval (red arrows). **(J)** Positive control for fosmid signal, on lymphoblastoid cell line m62. White arrows, normal copies of FBXL17.

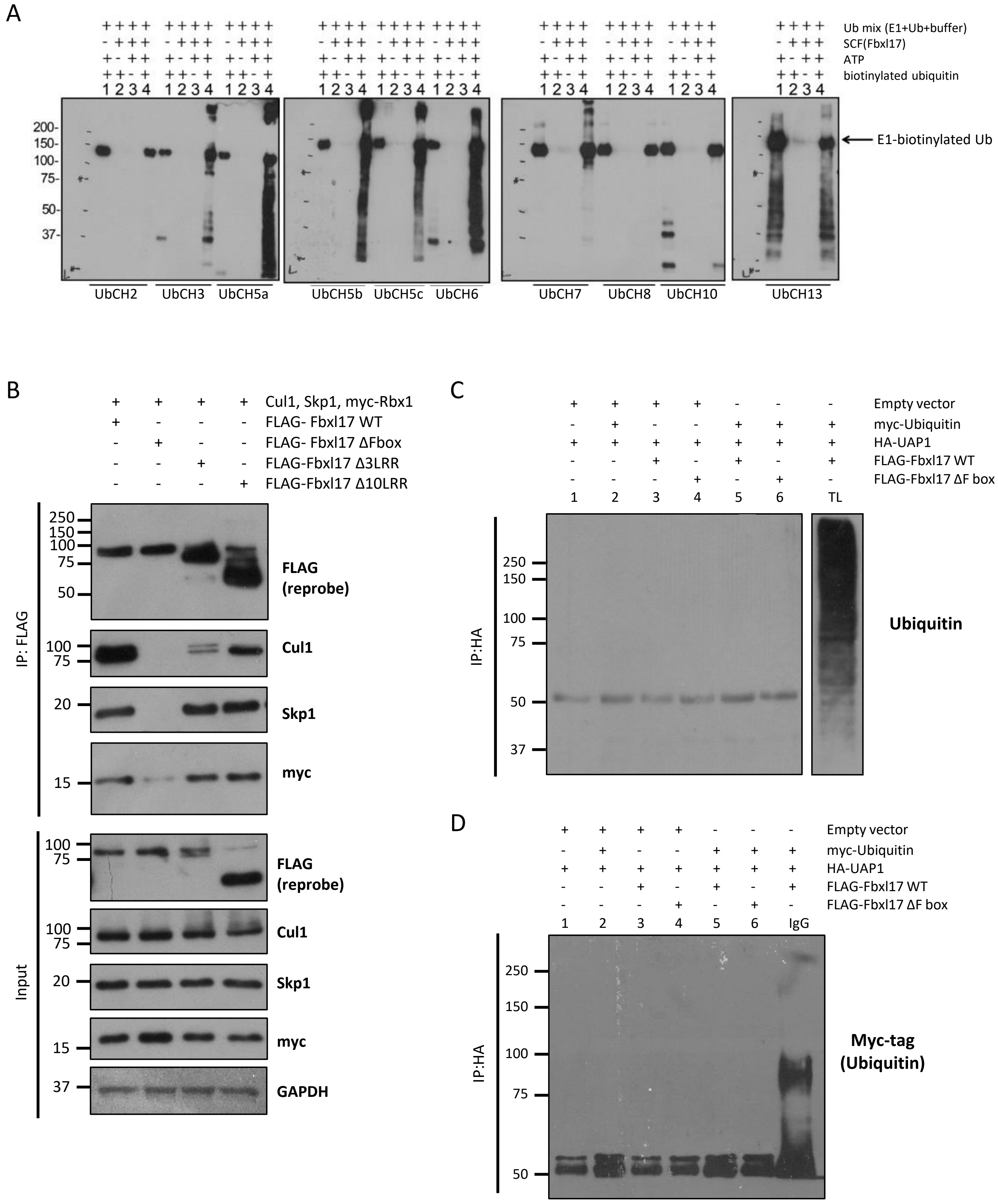

Supplementary Figure 2. (A) Screen of in vitro ubiquitination activity of purified SCF^{Fbxl17} complexes with various E2 ligases. Streptavidin-HRP was used to detect ubiquitinated proteins, n=2. **(B)** HEK293T cells expressing SCF components Cul1, Skp1 and Rbx1 and WT or mutant Fbxl17 constructs immunoprecipitated with anti-FLAG beads and immunoblotted as indicated. **(C)** . *In vivo* ubiquitination assay for UAP1. HA-UAP1 immunoprecipitated from HEK293T cells transfected with ubiquitin and indicated Fbxl17 constructs. Membranes probed with anti-Ub antibody, n=2. **(D)** as (C) but probed for myc-epitope tag.

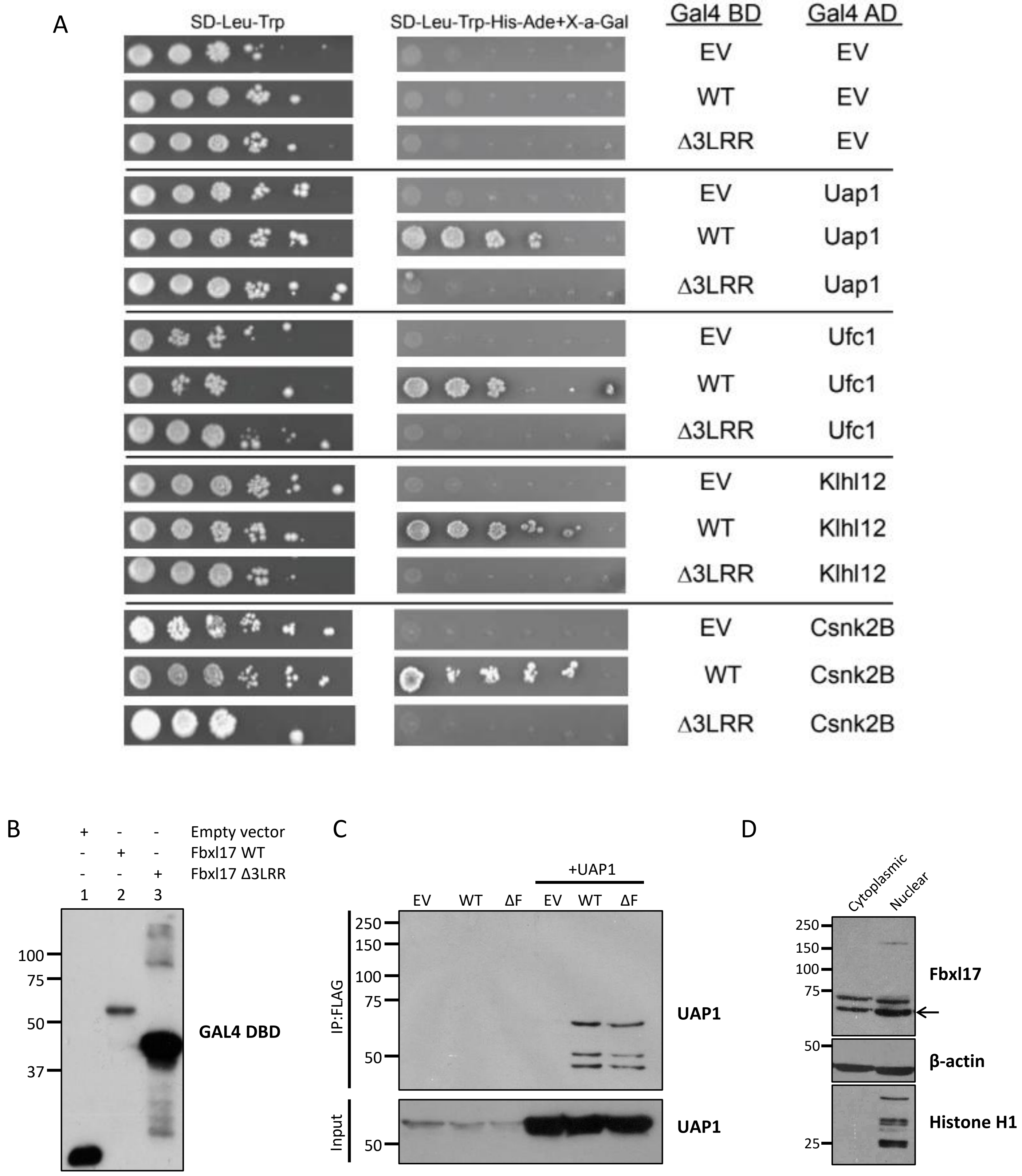

Supplementary Figure 3. (A) Binding between Fbxl17 (WT or Δ3LRR) and its interacting proteins, Uap1, Ufc1, Klhl12 or Csnk2B. Cloned cDNAs retransformed into yeast with Fbxl17 constructs. 10-fold serial dilutions on different selective media. **(B)** Bait expression in yeast. pGBKT7-FBXL17 and pGBKT7-FBXL17 (Δ3LRR) were expressed in AH109 yeast. Levels of Gal4 DNA-BD fused to Fbxl17 and Fbxl17 (Δ3LRR) were determined by immunoblotting analysis with GAL4 DBD antibody. **(C)** Immunoprecipitates using anti-FLAG beads from HEK293T cells expressing FLAG-Fbxl17 or FLAG-Fbxl17ΔFbox and Uap1, probed for Uap1, n=2. **(D)** Cellular fractionation of MCF7 cells immunoblotted for indicated proteins, n=2. Arrow represents bands corresponding to Fbxl17.

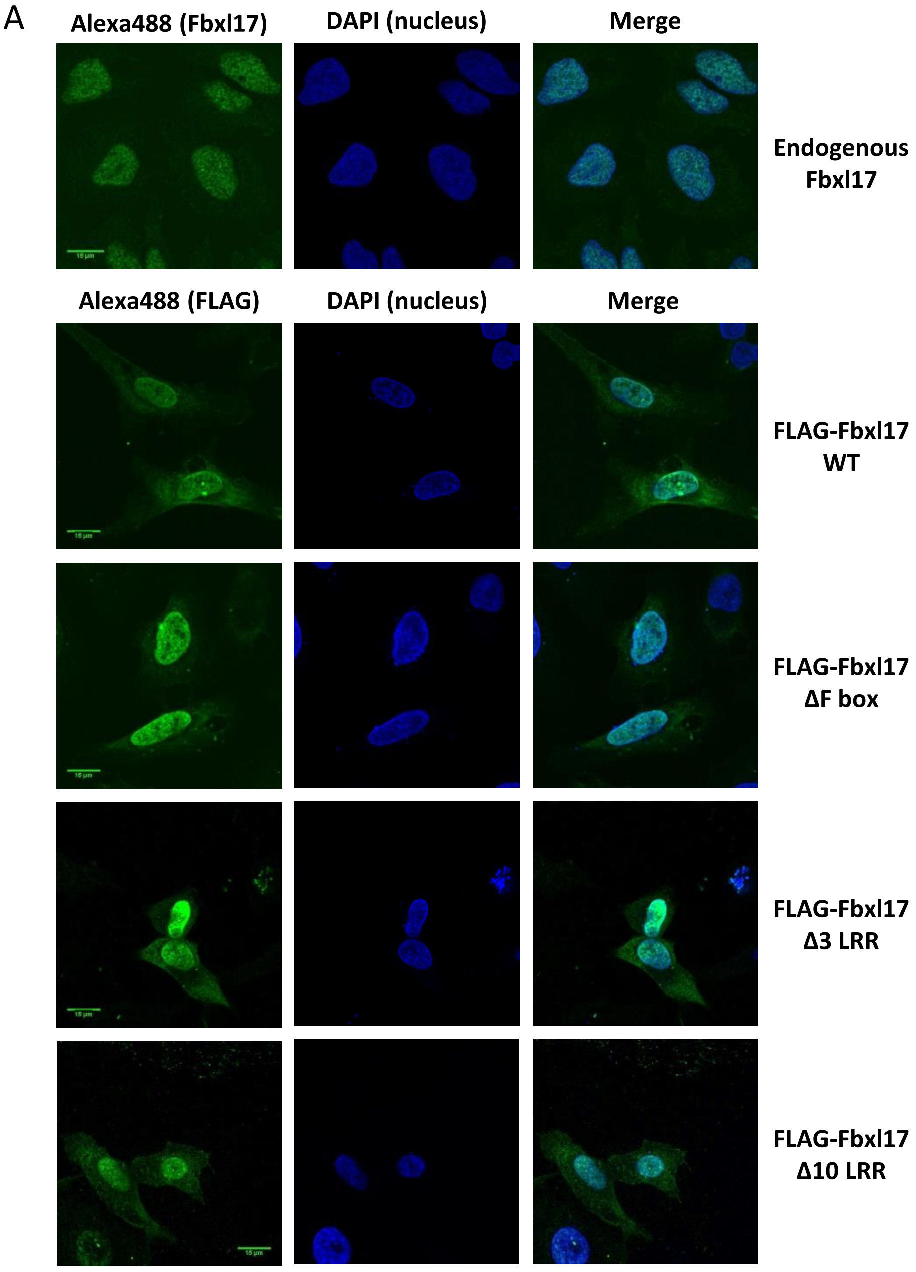

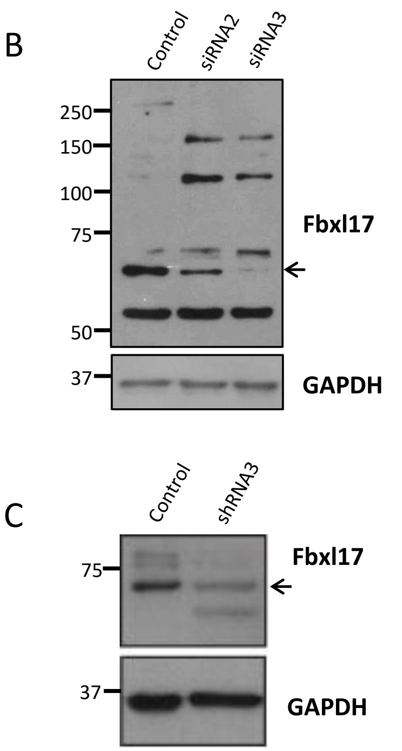

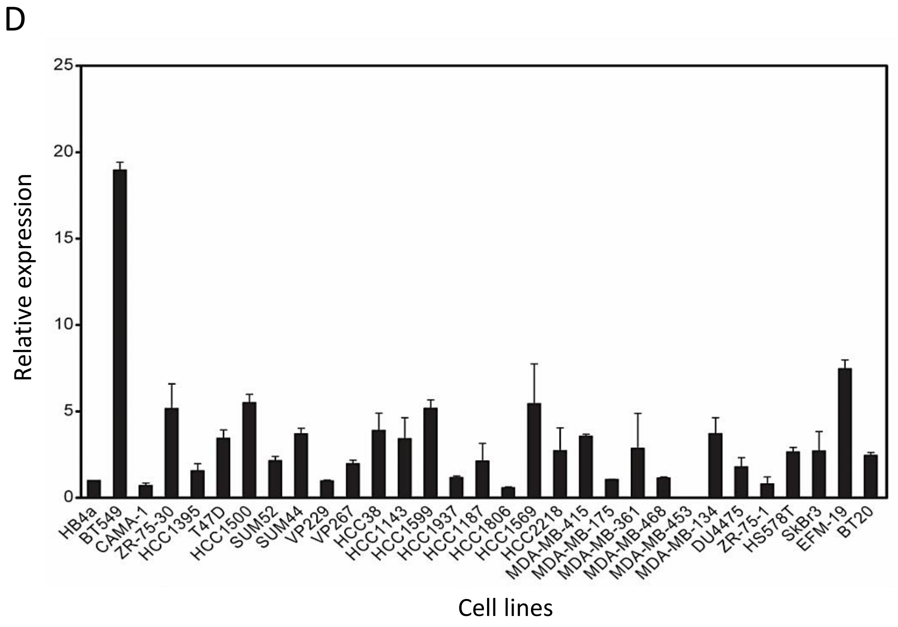

Supplementary Figure 4. (A) Subcellular localisation of endogenous Fbxl17 and indicated FLAG-Fbxl17 constructs using immunostaining with FBXL17/FLAG antibodies and Alexa Fluor 488. DNA visualized with DAPI, scale bar is 15µm. **(B)** Expression of Fbxl17 in U2OS cells treated with Fbxl17 targeting siRNA. Immunoblotted with anti-Fbxl17. Arrow represents band corresponding to Fbxl17. **(C)** Expression of Fbxl17 in HB4a cells expressing shRNA targeting Fbxl17 expression. Immunoblotted with anti-Fbxl17. Arrow represents band corresponding to Fbxl17. **(D)** Total RNA was harvested from a panel of normal breast and breast cancer cell lines. Levels of UAP1 mRNA were quantified by qPCR with primers located in exons 5 and 6 of UAP1 and normalised to GAPDH expression. Normalised UAP1 mRNA levels are presented relative to HB4a. Values represent mean ± s.d. of at least three independent experiments.

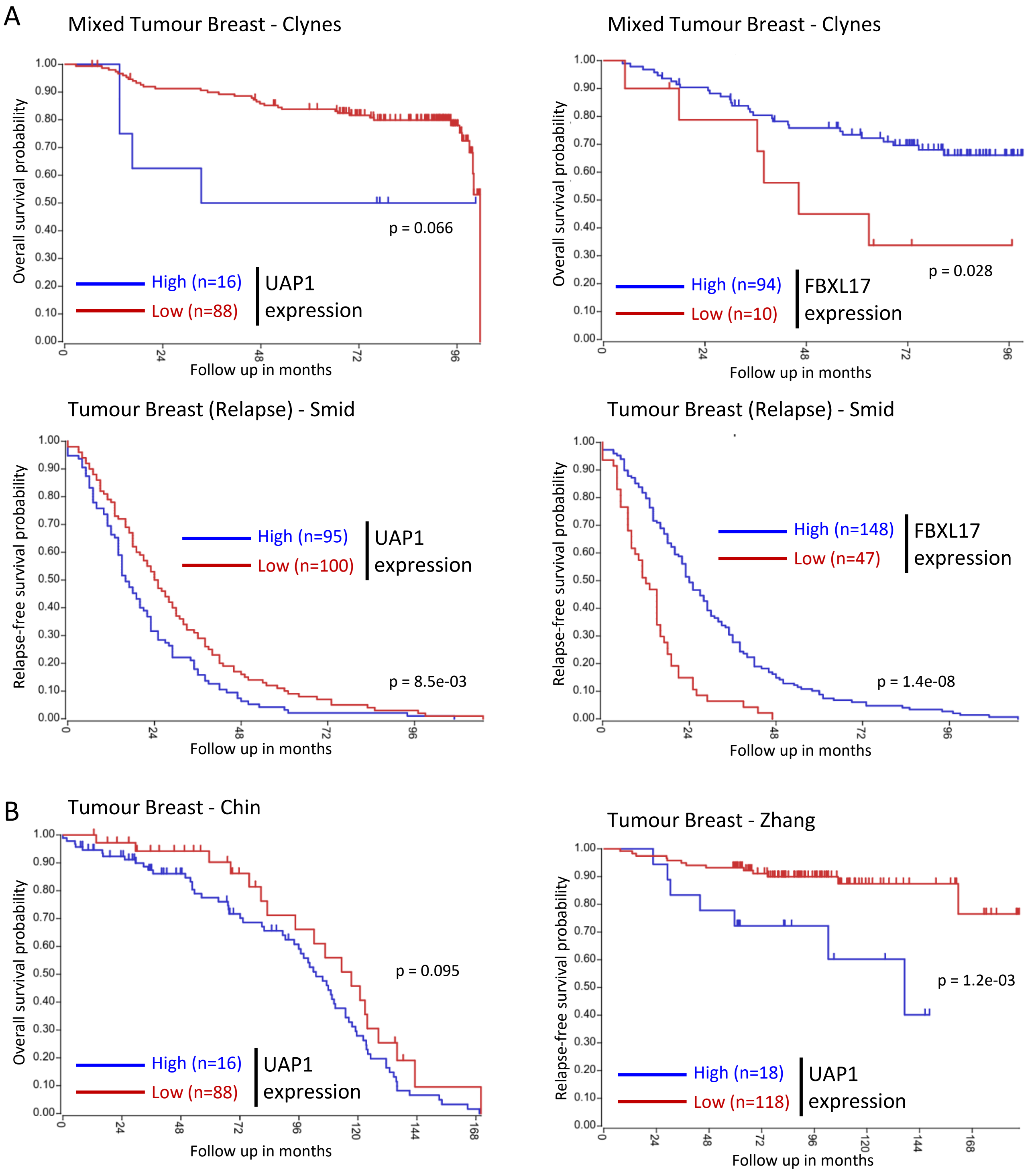

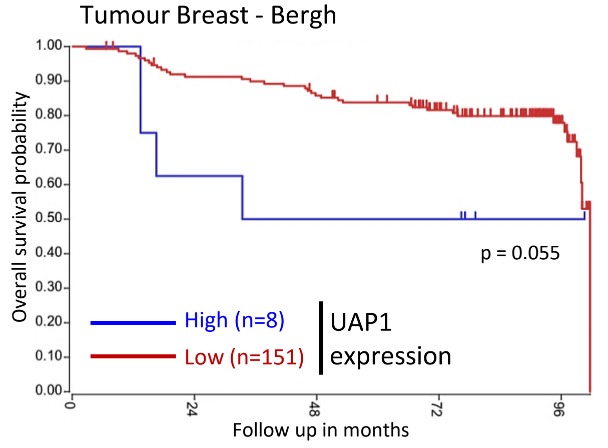

Supplementary Figure 5. Low Fbxl17 and high UAP1 expression levels correlate with reduced breast cancer survival. Kaplan Meier curves for gene expression were generated using the R2: microarray analysis and visualization platform (http://r2.amc.nl) resource. Publicly available breast cancer tumour datasets were mined for Fbxl17 and Uap1 expression data in correlation with survival probabilities.**(A)** Fbxl17 and Uap1 expression from the same datasets show high Uap1 and low Fbxl17 give a poorer prognosis for patient survival. **(B)** A further three datasets for which Fblx17 data was not available also show high Uap1 resulting in reduced survival.
